# The Concurrent Programming of Saccades

**DOI:** 10.1371/journal.pone.0168724

**Published:** 2016-12-22

**Authors:** Eugene McSorley, Rachel McCloy, Louis Williams

**Affiliations:** School of Psychology & Clinical Language Sciences, University of Reading, Reading, Berkshire, United Kingdom; University of Muenster, GERMANY

## Abstract

Sequences of saccades have been shown to be prepared concurrently however it remains unclear exactly what aspects of those saccades are programmed in parallel. To examine this participants were asked to make one or two target-driven saccades: a reflexive saccade; a voluntary saccade; a reflexive then a voluntary saccade; or vice versa. During the first response the position of a second target was manipulated. The new location of the second saccade target was found to impact on second saccade latencies and second saccade accuracy showing that some aspects of the second saccade program are prepared in parallel with the first. However, differences were found in the specific pattern of effects for each sequence type. These differences fit well within a general framework for saccade control in which a common priority map for saccade control is computed and the influence of saccade programs on one another depends not so much on the types of saccade being produced but rather on the rate at which their programs develop.

## Introduction

Eye movements made as part of our day-to-day activities are commonly made in sequences that serve to gather the information that allows us to achieve our goals in the most efficient and effective way possible [1, 2]. One of the most ubiquitous types of eye movement are those that move saccadically, rapidly shifting our point of regard from one location to another to overcome the natural acuity limits in our visual apparatus. These saccadic sequences must be translated into the underlying preparation and programming of sequential movements as individual saccades are, of course, made in a serial fashion, i.e., they are executed one at a time. Indeed there has been a great deal of research dedicated to understanding the control of isolated single saccadic eye movements and relatively little to their control when they form part of the sequences of multiple movements that occur during everyday tasks. It is essential to understand how multiple saccadic eye movements are programmed: are they, to some extent, planned in parallel, and if so which aspects of them are subject to parallel programming?

Behavioral and physiological studies have provided evidence indicating that the oculomotor (saccadic) system may be able to program at least two or three responses in parallel [3, 4, 5]. The parallel programming of multiple saccades has long been suggested [6], but many models of saccade generation are not designed to account for this as they are largely designed with single responses in mind. These adopt a “winner-take-all” approach in which a competition between different potential saccade targets coalesces around a single saccade response [7, 8]. Eye movements that take place during natural behaviors such as food preparation (sandwich making [9], tea making [10] or driving [11]) show complex scan paths. These paths contain many saccades to locations and objects that are only used in crucial actions at a later point in task completion suggesting the parallel use of information in order to sequence movements. In other everyday activities such as reading saccadic eye movements across the text suggest that word information (such as its visual features) maybe programmed in the parallel (see [12] for a recent review) and some successful models of reading incorporate elements of parallel processing in their architecture [13, 14]. In simpler laboratory based tasks, chains of multiple saccadic eye movements have been examined using corrective secondary saccades following error response [15, 16] or double or triple step tasks. The demand here is to make two or three saccades often as a response to a predetermined set of instructions (e.g., saccade to a target and then once more to the target two positions clockwise of it [17]; or execute two saccades when fixation is removed in response to a target which has stepped from location to another, [18, 19]). Alternatively sequences of saccades are made on the basis of a set of visual targets that are on screen at all times (box to box, to a set of green circles in amongst red ones, or directed by oriented Landolt C’s [20, 21, 22]) or ones that have been memorized such that subsequent saccades are memory-guided [21]. When considered across all of these studies, evidence consistently supports the position that information about saccade target locations beyond simply the next one is processed in parallel.

The evidence for parallel processing of saccades can take the form of performance enhancement or as an impact on the control of subsequent saccades. Improvements in performance in identification tasks have been found when targets (e.g., Gabor patches, [21]; letter identification [17]) are shown at future saccade target locations relative to other non-target locations. This has been interpreted as supporting a link between attentional enhancement at future saccade target locations and saccade programming such that attention is distributed in parallel along the sequence of saccade target locations. The extent of this is perhaps limited by task demands and the benefits have been shown to degrade the further along the sequence the future saccade target is from the current fixation position [17,20, 21]. The impact of task demands on saccade programming has also been reported in a series of studies by Vergilino-Perez with others [23, 24, 25, 26]. Their results, using strings of word like objects, have shown evidence that suggests that the parallel programming of double-step saccade sequences may take place in difference reference frameworks, either retinocentric or oculocentric. This depends on whether they are executed between- or within- object and if they are executed quickly.

Further evidence for the parallel programming of saccades has been shown in reports of corrective saccades following initial error responses. Very short latencies for corrective responses in the anti-saccade task have been found. In this task saccades are made in the direction opposite to a peripheral stimulus onset [27]. Sometimes (around 10–15%) erroneous reflexive saccades (‘pro-saccade errors’) are made to the peripheral stimulus and can be followed by a secondary corrective saccade after a very brief fixation period (0–100 ms) [28, 15, 29]). Very short interval corrective saccades have also been found in visual search paradigms after distractor directed saccades are corrected [30, 3, 16, 4, 5, 31]. These short fixation intervals prior to the corrective saccade have been taken as evidence for the parallel programming of two consecutive responses.

Additionally, evidence suggesting that saccades are programmed in parallel comes from the effect of second target location on metrics of first saccade response. Landing positions of first saccades are prone to averaging between target positions when two saccades are required with the second target location also influencing the trajectories of their movements [3, 18, 19]. Parallel programming of saccades is also suggested from a consideration of the effect of making sequences of saccades on the compression of visual space [32, 33]

Much like instances of corrective saccades with very short intervals, a common finding across many studies is of second or third saccade latency reductions (i.e., brief fixations or intersaccadic intervals), when multiple saccades are required [34, 35]. For example, Walker & McSorley [34] examined the parallel programming of saccades when they were made as part of a 2-step chain of either Voluntary then Reflexive saccade sequences or Reflexive then Voluntary saccade sequences. They found that the second saccade response, either reflexive or voluntary, was quicker than when either was executed in isolation. Walker & McSorley [34] interpret this as evidence that the cortical and subcortical maps involved in saccade generation function as a whole network, with the final selection of a unique saccade goal being performed on a common motor map [3, 4]. This is consistent with physiological observations made in Superior Colliculus [4, 36, 37].

Utilizing a similar paradigm to that employed by Walker & McSorley [34] the experiment presented here will further examine the parallel programming of multiple saccades. Specifically the aim here is to examine what aspects of saccades are being programmed in parallel when they are made as part of a 2-step sequence.

One possibility is that the spatial aspects of forthcoming planned saccades are being preprogrammed prior to their execution (e.g., the distance and direction of the next one or more saccades in a chain). On the other hand, there may be a general non-spatially specific readiness to execute saccades which manifests itself as a shortening of saccade response times when executing more than one saccade. Of course, these possibilities are not mutually exclusive. In order to examine this we will manipulate the position of the second target location during the execution of the first movement. If it is the case that spatial aspects of the second saccade are being programmed in parallel with the preparation and execution of the first saccade then shifting the location of the second target should result in interference with its execution. The pattern of this interference should reveal what spatial aspects, such as saccade direction or distance, are being programmed in parallel.

In this experiment, participants will be asked to make single voluntary saccades (i.e., internally generated saccadic eye movements made on the basis of a centrally presented symbolic directional arrow cue), single reflexive saccades (stimulus driven saccades made to a visual target on the basis of new information presented peripherally) or 2-step sequences consisting of chains of voluntary then reflexive or reflexive then voluntary saccades. On the basis of the research discussed previously, we would expect to see a speeding of the second saccadic response when compared with that response when made as a single movement (short inter-saccadic intervals) and an influence of the second target location on first saccade accuracy due to saccade averaging mechanisms [3,18,19]. First saccade latencies may be shorter but mixed findings have been reported with Experiment 1 of Walker & McSorley [34] showing a speeding of the first saccade response and their Experiment 2 showing no effect. In order to examine the question of what, if any, spatial aspects of the second target (if any) are being programmed in parallel the position of the second target was shifted during the execution of the first saccade response. This position shift could be towards or away from the first target location (distance manipulation) or its position was moved clockwise or counter clockwise of the first target location (direction manipulation). It constitutes new visual information about the location of the second saccade target and is bottom-up information that may be used to drive a new reflexive eye movement away from that driven by the original second saccade location. The idea is that the extent to which the new location of the second saccade target impacts on second saccade accuracy gives an index of its influence and should reveal the extent to which the spatial aspects of the second saccade program are programmed in parallel with the first.

## Method

### Observers

10 naïve observers participated in the experiment, aged between 18 and 51 years old. All had normal, or corrected to normal eyesight. The University of Reading Ethics Board approved the ethics of this study, and the study was conducted in accordance with the standards described in the 1964 Declaration of Helsinki. Participants provided written informed consent.

### Apparatus

Participants’ eye movements (left eye only) were recorded using an Eyelink II, which is a head mounted eye tracker with a 500 Hz sampling rate and a spatial resolution (RMS) of 0.025 deg. Participants placed their chin on a rest, which constrained any head movements and ensured the viewing distance remained at one meter. Before the experiment began, the eye tracker was calibrated using a 9-point grid, and then validated using a different grid. This was only accepted and the participant allowed to start the experiment when there was an average difference of less than 0.5 degrees between the actual eye position and that predicted from the calibration and the validation. Stimuli were presented on a 21” color monitor that had a refresh rate of 75 Hz.

### Stimuli

Fixation was an unfilled white square with a white “X’ in its center. Both voluntary and reflexive target stimuli were white circles (0.5 deg) overlaid with central black circles (0.25 deg). On each trial four voluntary targets were shown on the principal diagonals 8 degrees of visual angle from fixation. A single reflexive saccade target may also be presented. If present this flanked the voluntary saccade target 22.5 angular degrees in a clockwise or counter clockwise direction (3.1 degrees of visual angle). Stimuli were shown on a black background.

### Design

**Fig 1:**
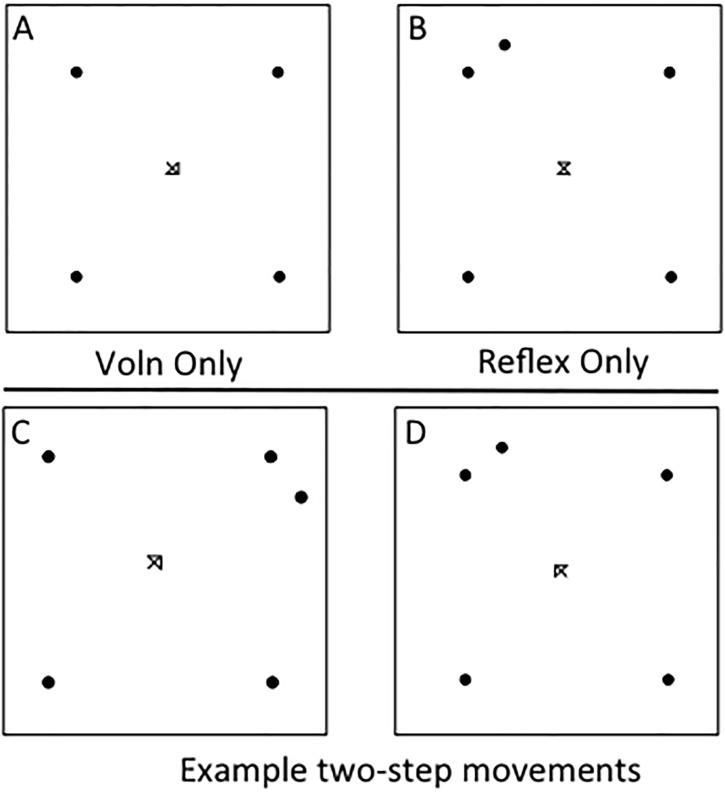
Examples of potential trial displays are shown. Saccade demand could be for a voluntary saccade only, a reflexive saccade only, a voluntary saccade followed by reflexive saccade or a reflexive saccade followed by a voluntary saccade. Instructions to participants were as follows: (A) if only an arrow appears make a response to the target it points at; (B) if only a new spot appears make a saccade to the spot; (C & D) if both an arrow and a new spot appear then execute two movements. In the VR block make a saccade to the spot indicated by the arrow first then a saccade to the new spot. In the RV block execute the movements in the other order: saccade to the new spot first then to the spot to which the arrow points.

Participants completed 220 trials in 2 counterbalanced blocks in which the saccade demand was either to move to one or two targets. In one block, if the trial demand was to execute sequential movements to two saccade targets, the participant was instructed to move to the voluntary saccade target first followed by the reflexive saccade target (VR), in the other block the instruction was to saccade to the reflexive target first followed by the voluntary target (RV). Voluntary saccade targets were indicated when two lines were removed from the fixation stimulus leaving an arrow pointing to one of the four possible voluntary saccade targets. Simultaneously with changes made to fixation, a reflexive saccade target was presented at one of the two locations flanking the indicated voluntary target (See Fig 1 for schematic example trial displays).

During each block some trials demanded a saccadic response to a single target. For single voluntary target responses two lines were removed from the fixation stimulus changing it to an arrow but no reflexive target was presented. For single reflexive target responses two lines were again removed from fixation but rather than a directional arrow stimulus this operation left an hourglass type figure (randomly standing upright or lying down on each trial). It is well documented that changes to stimuli at fixation have the effect of lengthening the latency of the first saccade response. Removing two lines from fixation for all trial types allowed the target to be identified with the same changes thereby ensuring that effects of this on saccade latency (in a visual sense e.g., luminance changes) would be same across both saccade types and trials demands.

**Fig 2:**
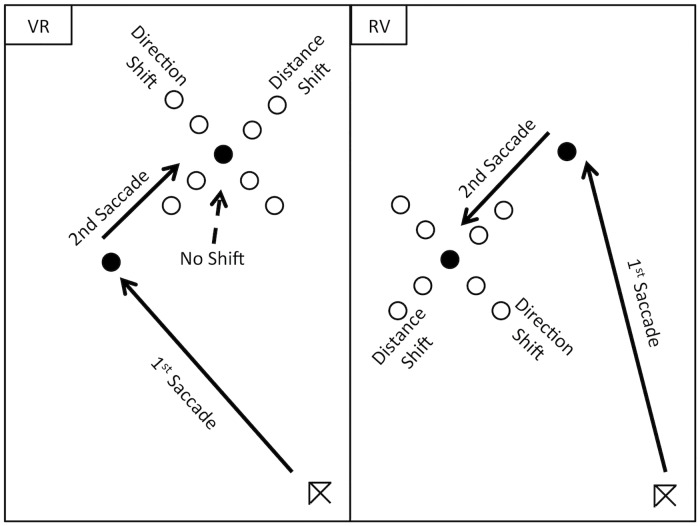
Shows schematic of the potential position shifts applied to the second target location (either no shift; a shift in distance or direction from 1st target location) made during first saccade execution. The potential position shifts for the second (Reflexive) target (including no shift) are shown on the left and the potential shift locations for the second (Voluntary) target are shown on the right (note the targets were black spots and remained so when their location was shifted. The shifted second target locations are depicted here as unfilled circles to allow their positions to be more easily discerned by the reader). Distance shifts were toward or away from the first target location (0.75 or 1.5 degrees of visual angle). Direction shifts were clockwise and counter clockwise to the first target location (10 and 20 angular degree shifts).

On a large proportion of trials (91%) the position of the second saccade target was moved during the first saccade response (defined as when the first saccade crossed an invisible boundary set at 4 degrees of visual angle from fixation). This took place in order to examine the programming of the second saccade target that was taking place during the preparation and execution of the first saccade movement. The shifts in position occurred during the first saccade response in order to take advantage of the reduction of visual sensitivity found during saccade suppression and thus minimize the disruption of new visual events on visual processing [38, 39]. This shift in position resulted in a change in the demand of the response to the second saccade target either in terms of its distance or angular deviation from the first saccade target position (see Fig 2). Changes in the distance of the second saccade target could be to one of four locations: 0.75 or 1.5 deg either towards or away from the first target. Changes in direction could also be to one of four locations: again 0.75 or 1.5 degrees of visual angle which translates to 10 or 20 angular degrees either clockwise or counter clockwise of the original second saccade target location (See Fig 2).

Overall this gave the following trial types:

Two types of single response trial types: (V) voluntary target response only, (R) reflexive target response only.

Two 2-step response trial types: (VR) response to voluntary then reflexive targets, (RV) response to reflexive then voluntary targets. With each VR and RV trial having one of the following position shifts applied to the location of the second target during the first saccade,

1. No shift
2. Four distance shift types in terms of degrees of visual angle of:
  -1.5 or -0.75 degs toward the first target location
  0.75 or 1.5 degs away from the first target location
3. Four direction shift types in terms of angular degrees relative to the first saccade target of:
  10 or 20 degs clockwise
  10 or 20 degs counter clockwise

Thus there were 11 possible trial types and for each trial type each participant completed 10 trials giving 110 trials per block and 220 in total.

### Procedure

Participants were first familiarized with the stimuli and the task, and were encouraged to carry out as many practice trials as they felt was necessary to become comfortable with the task and what they had to do. The calibration procedure was then carried out. Each trial started with the presentation of a centrally placed fixation stimulus and the four voluntary saccade target markers for 800–1200 ms, after which the experimental display was shown and consisted of either a change in fixation alone (an arrow indicating a single voluntary response) or a change in fixation (an arrow or hourglass figure) and the addition of a reflexive target (a single reflexive movement or a 2-step movement, VR or RV). After 1500 ms this was replaced by a blank screen for 500 ms.

### Data analysis

The eye tracking software includes a parser that was used to identify the start and ends of saccades using a 22 degree per second velocity and 8,000 degrees per second squared criteria (SR research Ltd). Further analysis of saccade latency and accuracy was accomplished offline using DataViewer (SR research Ltd) and in-house software analysis. Trials were only accepted as being “correct” if the saccade landed 2.5 degrees of visual angle from the target. If the trial demand was for two saccades then the trial was only accepted if two consecutive movements were executed each of which landed 2.5 degrees from the first and the second target. No other exclusion criteria were applied and resulted in a loss of 15% of trials (20% of the VR block and 10% of the RV block).

Saccade latency and accuracy are reported. First saccade latency was defined as the amount of time between the presentation of the experimental display and the initiation of the saccade while second saccade latency is the period of time between the end of the first saccade and the initiation of the second response. Accuracy was defined differently for trials in which distance or direction was shifted. The impact of shifts in second saccade target distance (-1.5, -0.75, 0, 0.75 and 1.5 degrees of visual angle position shifts) is simply shown by the effect on the second saccade amplitude. The impact of shifts in the second saccade target direction (10 and 20 angular degrees; with each shift collapsed across clockwise and counter clockwise directions) are shown by the difference between the angle of the second saccade relative to that required to land on the target (positive values are coded as being towards the location of the shifted second target position and negative away). Individual participant data is included in the Supporting information (S1 Table).

## Results

### Overall saccade latencies

**Fig 3:**
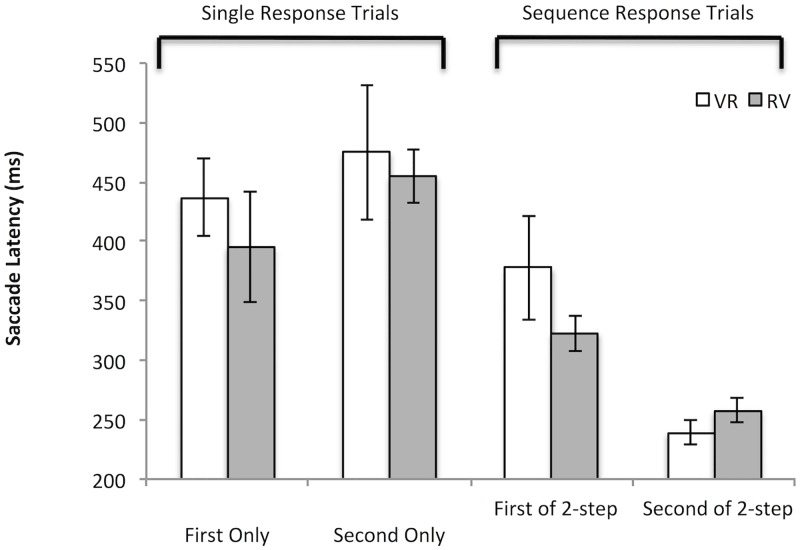
Shows average of median saccade latencies (ms) for voluntary and reflexive single target movements and when made as part of a 2-step movement (whether they could be considered the First or Second movement in the sequence order VR or RV). Saccade latencies recorded during voluntary then reflexive saccade target 2-step trials (VR) are shown as unfilled bars and those made during reflexive then voluntary trials (RV) are shown as Filled bars. To be clear, First Only refers to single responses made to the first target of the 2-step movement in that block (i.e., reflexive saccade in the RV block and voluntary saccade in the VR block), while Second Only refers to single responses made to the second target of the 2-step movement in that block (i.e., voluntary saccade in the RV block and reflexive saccade in the VR block). First of 2-step and Second of 2-step refers to the first response or the second response of the VR or RV 2-step trials depending on block. The saccade latencies executed during 2-step trials are collapsed across all the shifts in the position of the second target made during the first target response in order to examine the overall impact on saccade latency responses. Error bars are between participants’ error bars.

Fig 3 shows the average median saccade latencies for single and 2-step movements (collapsed across position shifts of the second saccade target). We carried out a three-way ANOVA with condition (VR or RV), sequence type (single or 2-step), saccade order (first or second) as factors. A significant effect of sequence type (F(1,9) = 73.234, MSE = 5442.642, p<0.001, η^2^ = .891) and a significant interaction of sequence of type and saccade order (F1,9) = 19.731, MSE = 5782.245, p = .002, η^2^ = .687) were found. All other comparisons were not significant (p’s>.280). To examine the overall benefit of making two movements versus a single movement, a comparison was made between the latencies of the first and second saccade response during the 2-step movements with that recorded when each saccade type is executed in isolation. For both VR movements and RV movements there was generally a significant, or at least a strong trend towards a, shortening of their latencies for both first and second saccades when they were being executed as part of a 2-step movement compared with when they were executed in isolation (1 tailed tests—VR: V alone vs. V1^st^ t(9) = 2.377, p = .041; R alone vs. R2^nd^ t(9) = 4.154; p = .002; RV: R alone vs. R1^st^ t(9) = 1.993, p = .077; V alone vs. V2^nd^ t(9) = 7.791, p < .002).

### Accuracy of the first saccade

**Fig 4:**
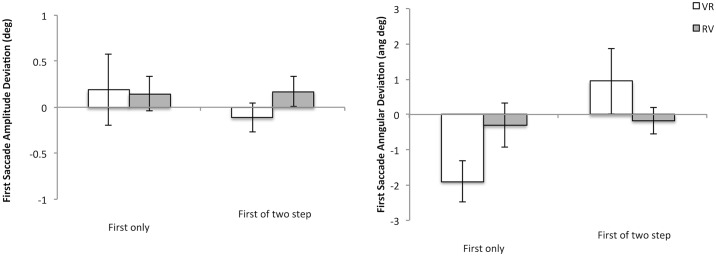
shows first saccade accuracy either in terms of its distance (amplitude of actual saccade from that required—shown on the left) or direction (angular deviation of actual saccade from that required to accurately land on the target—shown on the right) as a function of whether it was executed as part of a single movement (First only) or as a part of a sequence of two (First of 2-step). The VR sequence is shown as unfilled columns and the RV sequence as filled columns. Error bars are between participants’ error bars.

Fig 4 shows the accuracy of the first saccade response, whether made as part of a single step or part of a sequence of two movements, regardless of the shift in the position of the second saccade target (i.e., accuracy data for the first saccade made in the 2-step sequence is averaged across position shifts which occurred during first saccade execution). Accuracy is shown both in terms of the distance (left hand graph) of the first saccade or in terms of its direction (right hand graph). Two-way ANOVAs were carried out separately for distance and direction with Sequence Order (VR or RV) and Saccade Number (single movement only versus first saccade of a sequence of two) as factors. A significant main effect of the number of saccades made was found for the direction of the first saccade (F(1,9) = 5.972, MSE = 3.707, p = .037, η^2^ = .399). Further contrasts show that there was a significant difference in the VR sequence only (VR F(1,9) = 5.097, p = .05, η^2^ = .362; RV F<1) suggesting that when executing a VR sequence the first saccade direction is “pulled toward” the location of the second reflexive target. No effect was found on the saccade amplitude of the first movement whether executed singly or in consecutive pairs regardless of sequence order (all F’s<1).

### Latencies and accuracy of the second saccade

**Fig 5:**
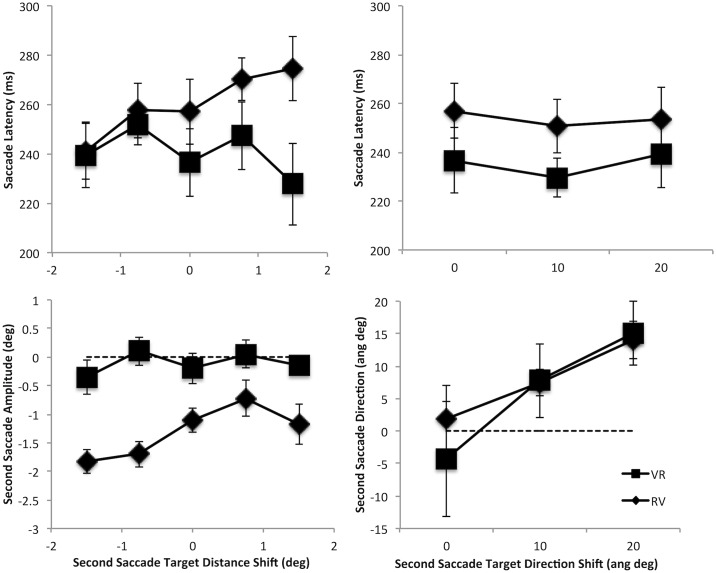
Shows the effect of the second saccade target position shift on second saccade target latency and accuracy. The effect of second saccade target position shifts in distance (towards or away from the first saccade target position with negative numbers showing the shift towards the first target location) is shown on the left and that from shifts in direction (shifts clockwise or counter clockwise of the first target position) is shown on the right. The upper row shows the average of the median saccade latencies (ms) for 2-step movements. The lower row shows second saccade accuracy in terms of the deviation from that required for the second saccade to land on the original second target location. For shifts in distance this is shown by the difference between the amplitude of the second saccade relative to that required to land on the original target location. For shifts in direction this is shown by the difference between the angle of the second saccade relative to that required to land on the target (positive values here are coded as being towards the location of the shifted second target position and negative away). Error bars are between participants’ error bars.

The impact of shifting the position of the second target location during the execution of the first saccade on second saccade latency is shown in the upper row of Fig 5. All location shifts show no change in the second saccade latency benefit reported in the *Overall Saccade Latencies* section (Fig 3) and hence no change in the magnitude of the parallel programming. This was confirmed with separate two-way ANOVA’s with sequence order (VR or RV) as one factor and either distance (5 levels: -1.5, -0.75, No Shift, 0.75, 1.5 degrees of visual angle) or direction (3 levels: No Shift, 10 or 20 angular degrees) as the other (all p’s>.164; although note that a trend analysis of RV latencies as function of distance reveals a marginal trend towards an effect: F(1, 9) = 4.334, MSE = 1.469.196; p = .067, η^2^ = .325).

The lower row of Fig 5 shows the impact on second saccade accuracy of shifting the position of the second target location during the execution of the first saccade. For changes made to the second target’s distance or to its direction from the first target location, the impact is shown by examining the second saccade amplitude or its direction relative to that required to land on the original second target location. As with the effect on second saccade latencies, the effects of shifts in the second target distance and direction were examined in separate two-way ANOVAs (i.e., Sequence Order by Distance; Sequence Order by Direction). The impact of shifts in distance on saccade amplitude shows main effects of both sequence order and distance, and an interaction between the two (Sequence Order F(1,9) = 104.952, MSE = 1.408, p < .0001, η^2^ = .921; Distance F(4,36) = 5.259, MSE = .334, p = .002; η^2^ = .369; Interaction F(4,36) = 2.778, MSE = .387, p = .041, η^2^ = .236). To explore this further one-way ANOVAs for each sequence order (VR and RV) were carried out with distance as a factor. For the VR sequence there was no effect of shifts in distance (F<1) but for the RV sequence there was a significant effect (F(4,36) = 13.019, MSE = .191, p < .0001, η^2^ = .591). Further planned contrasts for the RV sequence comparing each position shift vs. no shift separately, shows an effect at most positions (**-1.5:** F(1,9) = 10.359, MSE = .362, p = .011, η^2^ = .535; **-0.75:** F(1,9) = 6.409, MSE = .374, p = .032, η^2^ = .416; **0.75:** F(1,9) = 8.472, MSE = .439, p = .017, η^2^ = .485; **1.5:** F(1,9)<1). This shows that during execution of the VR saccade sequence shifts in the second target distance from the first target position had no effect on the second saccade but during the RV sequence second saccades were pulled towards the shifted second target location. The lack of an effect of the larger (1.5 deg) shift in position away from the first target location perhaps shows a spatial limit to this influence. This suggests that the second movement in the VR sequence is programmed wholly on the basis of the visual information presented prior to the execution of the first movement, while in the RV sequence this is not the case. In this sequence the metrics of the voluntary movement are influenced by the shifted location of the second target suggesting that the programming of the voluntary movement continues for longer than the reflexive movement in the VR sequence.

To examine the impact of shifts in the second saccade target direction clockwise and counterclockwise shifts have been collapsed (no significant interactions with direction were found) but the size of the direction shift, small and large, has been preserved. This is shown by the difference between the angle of the second saccade relative to that required to land on the target. Positive values are coded as towards the location of the shifted second target position and negative away. A two-way ANOVA with Sequence Order and Direction shows only a main effect of direction (F(2,18) = 8.038, MSE = 156.602, p = .003, η^2^ = .472; Sequence Order F(1,9)<1; Interaction F(2,18)<1) with larger direction deviations eliciting larger deviations in second saccade responses (**10 deg shift:** F(1,9) = 3.682, MSE = 426.105, p = .087, η^2^ = .290; **20 deg shift:** F(1,9) = 13.525, MSE = 370.514, p = .005, η^2^ = .600).

## Discussion

Here we examined which aspects of the second saccade program are being prepared in parallel with the first saccadic response. Participants were asked to make one or two voluntary and/or reflexive saccades on the basis of changes to the display. In most cases during a first saccade response the position of the second saccade target was shifted in distance or direction relative to the first target location. Both first and second saccade responses were found to show evidence supporting parallel programming. There was found to be a general speeding of the first and second saccadic response across sequence type and shift in second target location when compared with the saccade target response when made as a single movement. Further supporting evidence comes from effects of second saccade target on first and second saccade accuracy but the exact pattern of parallel programming was found to differ across sequence type.

### Latencies

Saccade latencies to the second saccade target were shorter than when an isolated single saccade was executed to that location. The requirement to make two movements also reduced the latency of the first saccadic movement. Furthermore, the reduction in the latencies of the first and second saccade was found not to be dependent on the order of saccade type. The finding that there are reductions in the response time of 2-step movements, compared to each executed in isolation, suggests that there is a benefit to knowing beforehand that two (or perhaps multiple) saccades are required in order to complete a task in that it reduces the overall time it takes to complete that task. This pattern of results is similar to that reported Walker & McSorley [34] who also found that second saccade latencies were shorter in VR and RV sequences. They can also be seen as part of the wider context of findings showing that saccades made as a part of a multi-step movement, either corrective movements or more deliberative ones, can be executed after short fixation periods [40, 3, 16, 4, 5, 35].

It is notable that the latencies of single step movements were longer than might be expected; this is especially noticeable for the reflexive saccades, which are much longer than the 150–250 ms response times often recorded. Very similar latencies to those found here were also reported by Walker & McSorley [34] where their task demands for VR and RV movements also produced longer response times for the single movements than would be expected ordinarily. They suggest that this may be due to a spillover from the underlying mechanisms involved in trials where 2-step movements are required on to trials were single responses are needed. These 2-step trials involve a combination of the predominantly top-down processes involved in voluntary saccade control and the largely bottom-up processes used to elicit reflexive saccades. As a result of both systems being active across trial types there is a general slowing of responses on the single saccade trials reported here compared with other experiments in which only single saccade responses are executed as part of the task. A second possibility is that the long latency of single saccade responses may also reflect the trial structure of the experiment, as there were a larger number of 2-step than single movement trials. Thirdly, and perhaps in tandem with this, the longer response times may reflect an uncertainty whether one or two responses were demanded and the longer than expected first saccade latencies may be due to participants holding on to single responses until it was clear that a second event was not going to demand two movements.

### Accuracy

The parallel programming of saccade sequences would also be expected to impact on both the first and second response accuracy. We found some evidence for this but notably there are differences across the two sequence types.

In terms of first saccade accuracy, the second saccade target in the VR sequences affects first saccade direction but not its amplitude. In contrast, RV sequences show neither effects on first saccade direction nor amplitude.

With regards to second saccade accuracy, changes in the position of the second saccade target affected the amplitude of the second saccade in the RV sequence but not for the VR sequence. Shorter or longer second voluntary saccade amplitudes were elicited as the second target was moved in distance towards or away from the first reflexive target location (although not for the largest shift outward, perhaps suggesting a spatial limit). Changes made in the direction of the second saccade target relative to the first saccade target were found to affect the response of the second saccade response for both the VR and RV sequences. Greater shifts in direction produced equivalently larger deviations towards the shifted target position for the both second voluntary and second reflexive saccades. This impact on saccade accuracy of a shift in the position of the second saccade target after first saccade execution shows that while the second saccade target may be programmed in parallel with the first it is by no means immune to the influence of new visual information.

### Sequence type

The specific pattern of effects for each sequence type can now be summarized and considered in turn (see Table 1):

**Table 1:** Shows effects of making sequence of two saccades. Greyed rows show RV sequences and white rows show VR sequences. 2-step first and second saccade latencies are compared with latencies for single saccade latencies with the second step latency effects shown regardless target shift (middle column) and as a function of the target shift (final column). 2-step first and second saccade accuracy is shown as a function of the direction needed to execute a correct movement. Note the blank middle column for accuracy was due to the effect of the second target position only being reported as a function of the shift in its position.

	First of 2-step	Second of 2-step (regardless of 2^nd^ target shift)	Second of 2-step (effect of 2^nd^ target shift)
**Latency**	R shorter (Fig 3)	V shorter (Fig 3)	V not affected (Fig 5)
	V shorter (Fig 3)	R shorter (Fig 3)	R shorter (Fig 5)
**Accuracy**	R not affected (Fig 4)	N/A	V distance and direction affected (Fig 5)
	V affected (Fig 4)	N/A	R distance affected not direction (Fig 5)

1. VR sequences: first responses are quicker and landing position is towards the second target position. Second saccade responses show no impact of changes in the second target location on their latencies: They were executed more quickly than single saccades regardless of the changes made during the first response. Changes made to the direction, but not the distance, of the second target was found to affect second saccade landing position.
2. RV sequences: first responses are quicker and landing position is not pulled towards the second target position. Second saccade responses show no impact of changes in the second target location on their latencies: They were executed more quickly than single saccades regardless of the changes made during the first response. Changes made to the direction and distance of the second target was found to affect second saccade landing position.

### A Common priority map

We can consider our results within a high-level general framework for understanding eye movement control. Converging evidence has suggested that there are three distinct but interconnected stages involved in the processing of goal directed saccades: A visual saliency stage in which bottom-up sensory encoding of stimuli takes place, the goal of which is to compute a saliency map [41]; an intermediate stage that combines saliency information with top-down goal demands and selection history to produce a common priority map of movement goals [42, 43]; and finally a motor stage on which motor representations are generated in order to produce eye movements. One interpretation of our results is that saccades are prepared and then pooled on the common priority map [3, 4, 42, 43] depending on its rate of development regardless of saccade type.

In terms of the effects of the second saccade program on first saccade response, it is known that voluntary saccades have a longer latency period prior to initiation than reflexive saccades [44]. This has been suggested to be due to different processing speeds involved in their generation. Reflexive saccades are generated more quickly largely through a corticotectal pathway from the parietal eye fields in IPS to SC to the brain stem generator. Voluntary saccades are generated more slowly as a result of programming occurring via pathways and structures more heavily located in the frontal lobe such as DLPFC, SEF and FEF [45]. Due to the temporal differences involved in their preparation it can be suggested they may co-occur on a common priority map at different states of activation such that a second saccade may be less well developed (less activation) in comparison with a more quickly developed (greater activation) first saccade. So under this explanatory framework and as shown in the results, a VR sequence would show a greater influence of the second reflexive movement on the first voluntary movement than that found for a RV sequence where there would be little effect of the slowly developing second voluntary saccade on the accuracy of the more rapidly developing first reflexive saccade.

Turning to the effects of second saccade target displacement shifts on the second saccade response, we can couch this within the same common priority map framework. When considering the speed at which the second saccade programs develop it is important to note that displacements of the second target location will be processed as new visual information as they introduce new bottom-up information to the display. This must then translate into a largely bottom up driven reflexive saccade. These shifts in second saccade target location change the nature of the 2-step sequences. Thus for both sequence types the original second saccade movement being programmed in parallel with the first is now being influenced by a new reflexive movement.

The VR sequence initially has a second reflexive saccade program. After a second saccade target shift, this is replaced with a new reflexive saccade target. However as the original R program is reasonably well developed and has an impact on first saccade response we would expect to see little effect of the shift in position on second saccade control and indeed this is what we find. The finding that there is an impact of direction shifts but not distance shifts may be taken as providing some support for this position, as it suggests only limited impact of the new reflexive target information on the original reflexive saccade program.

On the other hand, the RV sequence initially has a second voluntary saccade program. After the target shift this is also now replaced with a new reflexive saccade target. The more slowly developing voluntary saccade associated with the original second position was found to have little effect on the first reflexive response. However, it might be expected that the new more quickly developing second saccade driven by the new reflexive second saccade target would have an impact on the still developing original voluntary saccade program. This is indeed what we find. We show an effect on second saccade accuracy of shifts of both the distance and direction of the second target position that supports this suggestion. Overall, there is a larger influence of new reflexive information when this second saccade is originally a voluntary one than when it is reflexive.

Further support for this interpretation comes from an examination of the relationship between second saccade latency and its accuracy. If there was a direct relationship between the speed that a saccade was processed and its accuracy then there should be an improvement of second saccade accuracy towards the shifted target position as second saccade latency increases regardless of saccade sequence type. A correlation between second saccade accuracy and its latency across sequence type showed a positive relationship in that longer second saccade latencies resulted in more accurate saccades both in terms of amplitude shifts and direction shifts (Amplitude R = .152, p = .001; Direction R = .223, p < .001).

## Conclusion

We have reported a pattern of parallel programming that fits well within a general framework [36, 37] for saccade control in which a common priority map for saccade control is computed. Reflexive saccades develop quickly via a bottom-up visual stage in which the computation of visual saliency takes place (perhaps by a corticotectal route) while voluntary saccades are programmed by a more time consuming top-down goal selection stage (a more frontally driven network involving DLPFC, SC, SEF and FEF). This combines with saliency to produce a priority map which then feeds down to a movement stage in which the motor representations are generated that ultimately move the eyes. In such a framework sequential saccadic eye movements are partially programmed in parallel and combined with new information processed on the fly. The influence of saccade programs on one another depends not so much on the types of saccade being produced but rather is dependent on the rate at which their programs develop. More quickly developing future saccade programs will have greater influence on preceding movements. Our findings fit well with previous reports showing that when multiple saccades are made as part of task demands or as a corrective movement following an erroneous response they are programmed in parallel. The evidence in favor of parallel saccade programming shows performance enhancement [17] at future locations of saccade targets compared other potential target locations; very short second saccade latency periods either in comparison to latencies usually found when executing isolated saccades [4, 5] or when directly compared to saccades on a like by like basis [34]. There has also found to be an impact from a second saccade target location on the first saccade landing position and its trajectory [18]. Furthermore, findings from within and between word/object saccade sequences show a similar flexibility in saccade parallel programming to that reported here, also with a dependency upon saccade latency [46, 47, 48, 49]. It is interesting to note in the context of visual space compression [32, 33] that our results would suggest that the compression of visual space around the onset of the second saccade would only be found, or rather be found more strongly, in trials with greater evidence of parallel programming [but see 50]. This would be expected to occur in the RV sequences but more specifically we would suggest that the extent of visual space compression would increase on a trial-by-trial basis depending on the extent of parallel programming.

## Supporting Information

S1 Table (XLSX)
